# Case of Stone in a Girl Relieved by Lithontritie

**Published:** 1840

**Authors:** 


*Case of Stone in a Girl relieved by Lithontrite.*—Rachel, an orphan child, aged ten years, came in laboring under calculus in the bladder. Her general health had been for some time deranged. The mucous membranes generally were the seat of irritation. Jacobson's lithontrite was introduced and the stone readily seized. The crash of the stone was heard through the theatre. A portion of it came away at the time, and the rest of it in a few days. She had no return of stone, and became robust and hearty.

I am sorry to say this poor little girl died a few days since at the Asylum, of bronchial ulceration and general mucous irritation.—*Ib.*

